# Exposure of environmental Bisphenol A in relation to routine sperm parameters and sperm movement characteristics among fertile men

**DOI:** 10.1038/s41598-018-35787-5

**Published:** 2018-12-03

**Authors:** Honglei Ji, Maohua Miao, Hong Liang, Huijuan Shi, Dasheng Ruan, Yongbo Li, Jian Wang, Wei Yuan

**Affiliations:** 1 Department of Epidemiology and Social Science, NHC Key Lab. of Reproduction Regulation (Shanghai Institute of Planned Parenthood Research), Fudan University, Shanghai, China; 2 NHC Key Lab. of Reproduction Regulation (Shanghai Institute of Planned Parenthood Research), Fudan University, Shanghai, China; 3 National Chemical Low Carbon Technology and Engineering Center, Kunshan, Jiangsu China; 4 Population and Family Planning Institute of Guizhou Province, Guiyang, Guizhou China

## Abstract

Although several human studies have examined bisphenol A (BPA) exposure in relation to routine sperm parameters, evidence of BPA’s effects on sperm movement characteristics is limited. We examined associations of BPA exposure with sperm parameters including sperm movement characteristics among fertile men. The cross-sectional study was conducted in Sandu County, Guizhou Province, China. Subjects provided semen samples analyzed by computer-aided sperm analysis (CASA) system and urine samples for BPA assay. They were invited to complete an in-person interview with a structured questionnaire to obtain demographics, lifestyle factors, etc. In final analyses, 500 subjects were included. We used multivariate linear regression analyses to estimate associations between BPA and sperm parameters after adjusting for potential confounders. BPA was detected in 73.6% of urine samples, with a geometric mean of 0.44 μg/gCreatinine. Compared with subjects of undetected BPA, subjects with detected BPA had increased Linearity (LIN, β: 2.19, 95% confidence interval (CI): 0.37, 4.0), Straightness (STR, β: 1.47, 95% CI: 0.19, 2.75), Wobble (WOB, β: 1.75, 95% CI: 0.26, 3.25), reduced Amplitude of lateral head displacement (ALH, β: −0.26, 95% CI: −0.5, −0.02) and Mean angular displacement (MAD, β: −2.17, 95% CI: −4.22, −0.11). Subjects in the highest tertile of creatinine-adjusted BPA group had lower sperm concentration than those with undetected BPA. Dose-response relationships of BPA with LIN, WOB, ALH, MAD and sperm concentration were demonstrated by statistically significant trends across tertiles of creatinine-adjusted BPA concentrations. Similar results were obtained using unadjusted BPA concentrations. Exposure to environmental BPA would decrease sperm concentration and sperm swing characteristics (ALH and MAD), and increase sperm velocity ratios (LIN, STR and WOB), which might mediate further effects on impaired male fecundity.

## Introduction

Bisphenol A (BPA) is widely recognized as one of the endocrine disrupting chemicals (EDCs), and used in a variety of common consumer products. Most notably, BPA is present in polycarbonate plastics, the epoxy resin liners of aluminum cans, and thermal receipts^1^. BPA can be leach from BPA-containing products into food, water, and ecosystems^2^, leading to widespread human exposure. The detection rate of urinary BPA in Chinese population was reported to range from 50% to 100% across different regions of China^3–5^.

The endocrine-disrupting properties of BPA have largely been demonstrated, that BPA has exhibited both estrogenic and antiandrogenic effects^6^. Accumulating experimental studies have revealed that BPA can bind steroid receptors, decrease steroidogenic enzymes, and produce reactive oxygen species (ROS)^7–10^, which may interfere with spermatogenesis. Rodent studies of both low- and high-dose BPA exposure have reported reductions of sperm count and testosterone level, impairment of sperm motility, and sperm DNA damage^11–15^.

Human studies on the effects of exposure to BPA on sperm parameters are limited and inconclusive. Studies of occupationally exposed men^16^ and men recruited from an infertility clinic^17^ reported that urinary BPA concentration was associated with decreased sperm count and motility. In studies of general populations^18,19^ or fertile men^20^, BPA concentration was associated with decreased sperm concentration and motility, although most of the associations were not statistically significant. However, studies on relationships of urinary BPA levels with sperm movement characteristics have not been well documented yet. It is well acknowledged that sperm movement characteristics can predict fertilizing potentials of spermatozoa^21–26^. In general, spermatozoa with higher velocities, lower Linearity (LIN) and bigger Amplitude of lateral head displacement (ALH) possess better fertilizing potentials. So far, to our knowledge, only two studies^17,19^ investigated sperm movement characteristics measured with computer-aided sperm analysis (CASA) system in relation to BPA exposure. However, both studies have methodological flaws, such as recruiting subjects from infertility clinics or assessing sperm movement characteristics the next day after semen collection.

The aim of the present study was to examine the associations of urinary BPA concentrations to both routine sperm parameters and sperm movement characteristics measured by CASA system among fertile men in China.

## Methods

### Study population

We conducted a cross-sectional study based on a primary health program that aimed to promote reproductive health of couples in less developed areas by providing free medical counseling as well as free semen quality assays for couples of childbearing age. The study was conducted from July to August, 2012 in Sandu County (Autonomous County of Shui nationality), Guizhou Province, China. Inclusion criteria included males, 18–55 years and having at least one child. The men who (1) had been diagnosed with disease of urogenital system including testis, epididymis and prostate, (2) had severe heart, liver, lung, kidney, endocrine diseases or (3) any history of hospital diagnosed mental illness were excluded from the study.

A total of 774 eligible men, corresponding to a participation rate of 75%, agreed to participate. Among them, 507 men provided both semen samples and urine samples for BPA assay. We further excluded 7 men who did not provide any in-person information. Finally, 500 men (64.6% of eligible males) were included in the study. None of the participants or their wives were received any medical interventions, and 66.36%, 19.72%, 7.89% and 6.03% conceived the first child within 6 months, 6–12 months, 12–24 months and greater than 24 months, respectively.

### In-person interview and bio-sample collections

The participants were invited to local clinics and interviewed with structured questionnaires. We obtained information on demographics, lifestyle factors (smoking and alcohol intake), history of exposure to pesticides, occupational exposure to high temperature, and reproductive history. Individuals who reported smoking at least one cigarette per day for the last six months were considered as smokers. Individuals who reported drinking alcoholic beverage (wine, beer, or liquor) at least once a week for the last six months were considered as drinkers. History of exposure to pesticides was defined as contacting any pesticides at least half an hour each day for at least 7 days each year. Individuals who reported to be workers of casting, smelting or bread baking were considered as occupational exposure to high temperature. Body height and weight were measured with barefoot and clad in light underwear, following the recommendations of the Committee on Nutritional Anthropometry of National Health and Nutrition Examination Survey. Body mass index (BMI) was calculated as weight (kg) divided by squared height (m^2^).

A single-spot urine sample was collected from each subject in a polypropylene cup (BPA free) when they visited the clinic for interview. The urine was frozen without preservatives and stored at −20 °C in local clinics within 14 days before they were shipped on dry ice by plane to the laboratory at the Shanghai Institute of Planned Parenthood Research (Shanghai, China) and stored at −80 °C for further analysis. A single semen sample was also, at the same time, collected by masturbation into 25-ml sterile polystyrene jars (BPA free) from each participant.

### Semen analysis

The semen samples were analyzed within 1 hour following ejaculation. Semen analyses were conducted after liquefaction at the clinics by WLJY-9000 CASA system (Beijing Weili New Century Science & Tech Dev. Co. Ltd., Beijing, China)^27^. Main technical parameters are as follows: image acquisition frame: low and middle sperm concentration collected at 20 Hz, while high sperm concentration at 7 Hz; acquisition interval: 3 ms; maximum sperm motile velocity: 200 μm/s; area range of spermatozoa head detected at 7–60 μm^2^. Features of system software: adjusting greyscale threshold, collecting spermatozoa and excluding nonsperm granules.

The same professional technician performed semen analysis for all specimens according to World Health Organization (WHO) guidelines and instruction of the instrument manufacturer. Briefly, to measure both sperm concentration and motility, 5 μL of semen from each sample was placed into a pre-warmed (37 °C) 10 um-depth MAcro. counting chamber provided by the instrument manufacturer. A minimum of 200 sperm cells from at least four different fields were analyzed from each specimen. Sperm concentration, total count, forward mobility, and nine parameters of sperm movement characteristics were measured to profile men’s sperm quality. Total sperm count (10^6^) was calculated by multiplying sperm concentration (10^6^/ml) by semen sample volume (ml). Motile sperm was defined as WHO grade “a” sperm (rapidly progressive with a velocity ≥ 25 μm/s at 37 °C) plus “b” grade sperm (slow/sluggish progressive with a velocity ≥ 5 m/s but <25 μm/s). Sperm movement characteristics are composed of three indicators on sperm motion velocity (Curvilinear velocity (VCL), μm/s; Straight-line velocity (VSL), μm/s; Average path velocity (VAP), μm/s), three on velocity ratio (Linearity (LIN) equals to VSL/VCL; Straightness (STR) equals to VSL/VAP; Wobble (WOB) equals to VAP/VCL), and three on sperm swing characteristics (Amplitude of lateral head displacement (ALH), μm; Mean angular displacement (MAD), degree; Beat-cross frequency (BCF), Hz)^28^.

### Urinary BPA measurements

The urine samples were analyzed at the collaborative laboratory of East China University of Science and Technology (Shanghai, China). Urinary concentrations of total BPA (free plus conjugated species) were measured by modified high-performance liquid chromatography (HPLC) as described previously^3^. In brief, urine samples were treated with 100 μL phosphorous acid buffer of 0.01 mol/L (PH = 5.0) and 20 μL β-glucuronidase (Sigma) for hydrolyzation, and were subsequently extracted twice using ether (HPLC grade, Dikma). We collected and evaporated the supernatants using nitrogen gas. The residue was dissolved in 60% acetonitrile (HPLC grade, Dikma) and analyzed using HPLC equipment on the following parameters: column, Inertsil ODS-3, 4.6 mm × 250 mm, 5 mm; mobile phase A and B, acetonitrile/water (40:60, v/v), equivalent grade; flow: 1.0 mL/min; FLD, excitation wavelength 275 nm, emission wavelength 300 nm. Water used in the assay was from Millipore Super-Q Plus water purification system (Bedford, MA).

Glass-made analytic instruments were used throughout entire analytic procedure to avoid possible contamination. Besides, blank samples, which used purified water instead of urine, were conducted randomly during each HPLC analysis to confirm an absence of BPA contamination. The BPA fraction was confirmed by the standard BPA (HPLC grade, Shanghai Yuanxing Company) with the same HPLC base. The relative standard deviation (RSD) of replicate analysis of samples was less than 5.26%. Absolute recoveries of BPA ranged from 83.7% to 98.7%.

The limit of detection (LOD) of BPA in this study was 0.12 μg/L, which was comparable to those reported at 0.1–0.4 μg/L in some studies^29–31^. BPA levels below the LOD were assigned a value of LOD divided by the square root of 2, based on a conventionally accepted practice^32^. Adjustment for creatinine was performed to account for urine volume, which was applied in all analyses.

### Statistical analysis

Descriptive statistics of demographic characteristics, urinary BPA concentration and semen parameters were tabulated. Due to skewed distributions, sperm concentration and total count were natural logarithm (ln) transformed. Linear regression models were used to estimate associations of sperm parameters with BPA exposure. BPA exposure was first dichotomized as undetected and detected groups and differences of sperm parameters between the two groups were estimated. Those with detected BPA were further categorized into 3 groups according to tertiles and associations of sperm parameters with different BPA terties were examined with subjects with undetected BPA as reference group. The associations between BPA and sperm parameters were examined by introducing BPA categories as a discrete variable in the linear regression models. To test the dose-response pattern of the association, we repeated the analysis by introducing BPA categories as a continuous variable (0–3).

We adjusted for covariates, including age (<25, 25–29, 30–34, 35–39 and ≥40 years), education (≤primary school, junior high school and ≥senior high school), ethnicity (Shui, Bouyei, Miao, and other nationalities), smoking (Yes, No), alcohol intake (Yes, No), abstinence period (<2, 2–7 and >7days), BMI (<18.5, 18.5–24.9 and ≥25 kg/m^2^), history of exposure to pesticides (Yes, No), and occupational exposure to high temperature (Yes, No).

We re-examined the relationships between BPA exposure and sperm parameters using unadjusted BPA concentrations of all participants (shown in Supplemental Material, Table S1). The relatively large study sample recruited from communities made it almost inevitable to cause discrepancies in time of urine collection and abstinence periods. Thus, sub-group analyses by time of urine collection and abstinence periods were considered a priori. In order to reduce the misclassification of BPA level resulting from a large discrepancy in time of urine collection, we restricted analyses to the subjects whose urine samples were collected between 9 to 11 am (showed in Supplemental Material, Table S2). We also restricted analyses to the participants whose semen samples were obtained within an abstinence period of 2 to 7 days, which would reduce the misclassification of semen measures with duration of abstinence.

All data handling and statistical analyses were performed in SAS version 9.4 (SAS Institute, Inc., Cary, North Carolina).

### Ethics

The study was reviewed and approved by the ethics committee board of Shanghai Institute of Planned Parenthood Research (IRB00008297). All participants gave written informed consent before engaging in the study. We confirm that all methods were performed in accordance with the relevant guidelines and regulations.

## Results

### Characteristics of the study population and distribution of BPA

BPA detection rates and creatinine-adjusted BPA concentrations by the characteristics of the study population are presented in Table 1. Urinary BPA was detected in 73.6% subjects. Geometric mean (SD) of unadjusted and creatinine-adjusted BPA concentration was 0.38 (4.74) μg/L and 0.44 (5.33) μg/gCr. The medians (5^th^–95^th^ percentiles) of unadjusted and adjusted BPA concentrations were 0.32 (0.08–6.86) μg/L and 0.35 (0.05–8.19) μg/gCr, respectively. BPA concentrations tended to be higher in subjects who were Bouyei nationality, with abstinence period of less than 2 days, overweight and whose wives were less than 30 years old.

**Table 1:** Characteristics of the study population and distribution of BPA (n = 500).

Characteristics	N (%)	BPA detection rate		Creatinine-adjusted BPA(μg/gCr)	
		N (%)	*p*-value	Geometric mean (SD)	*p*-value
All subjects	500 (100)	368 (73.6%)		0.44 (5.33)	
Age group (years)			0.1290		0.8116
<25	65 (13.0)	51 (78.46)		0.48 (4.74)	
25–29	145 (29.0)	112 (77.24)		0.42 (5.64)	
30–34	135 (27.0)	103 (76.3)		0.47 (4.84)	
35–39	94 (18.8)	62 (65.96)		0.37 (5.14)	
≥40	61 (12.2)	40 (65.57)		0.51 (7.05)	
Education			0.6174		0.4525
≤primary school	178 (35.6)	127 (71.35)		0.44 (4.99)	
Junior high school	252 (50.4)	187 (74.21)		0.47 (5.76)	
≥Senior high school	70 (14.0)	54 (77.14)		0.35 (4.76)	
Race/ethnicity			0.8995		0.0004
Shui nationality	250 (50.92)	181 (72.4)		0.35 (4.92)	
Bouyei nationality	133 (27.09)	98 (73.68)		0.73 (6.62)	
Miao nationality	88 (17.92)	67 (76.14)		0.35 (3.79)	
Other nationalities	20 (4.07)	14 (70.0)		0.69 (5.99)	
Smoking			0.3798		0.1266
No	192 (38.48)	137 (71.35)		0.38 (4.88)	
Yes	307 (61.52)	230 (74.92)		0.49 (5.64)	
Alcohol intake			0.5759		0.1169
No	170 (34.84)	128 (75.29)		0.52 (5.43)	
Yes	318 (65.16)	232 (72.96)		0.4 (5.18)	
Abstinence (days)			0.3292		0.1734
<2	114 (22.89)	88 (77.19)		0.55 (5.61)	
2–7	282 (56.63)	200 (70.92)		0.44 (5.47)	
>7	102 (20.48)	78 (76.47)		0.36 (4.66)	
BMI			0.6671		0.1284
<18.5	23 (4.67)	16 (69.57)		0.29 (5.69)	
18.5–24.9	379 (76.88)	282 (74.41)		0.42 (5.73)	
≥25	91 (18.46)	64 (70.33)		0.59 (8.01)	
History of pesticide usage	0.2178		0.6904		
No	277 (55.73)	210 (75.81)		0.46 (5.02)	
Yes	220 (44.27)	156 (70.91)		0.43 (5.72)	
occupational exposure to high temperature		0.9628		0.7836	
No	475 (95.38)	349 (73.47)		0.44 (5.32)	
Yes	23 (4.62)	17 (73.91)		0.39 (4.74)	
**Demographics of wives**					
Female age group (years)			0.0220		0.9873
<25	139 (30.68)	106 (76.26)		0.44 (5.55)	
25–29	120 (26.49)	100 (83.33)		0.45 (4.41)	
30–34	106 (23.40)	73 (68.87)		0.40 (5.47)	
35–39	51 (11.26)	32 (62.75)		0.44 (6.28)	
≥40	37 (8.17)	25 (67.57)		0.45 (7.02)	
Female education			0.4961		0.6306
≤primary school	188 (41.69)	134 (71.28)		0.39 (4.63)	
Junior high school	235 (52.11)	178 (75.74)		0.45 (6.04)	
≥Senior high school	28 (6.21)	22 (78.57)		0.50 (4.90)	

There are 9, 1, 12, 2, 7, 3, 2, 47 and 49 missing values in race, smoke, alcohol intake, abstinence period, BMI, history of pesticide usage, occupational exposure to high temperature, female age and female education respectively; There are 52 missing values in creatinine measurements totally.

### Distribution of sperm parameters

Table 2 shows the descriptive data on sperm parameters. Sperm concentration, total count, and forward motility in 6.01%, 36.96% and 30.43% semen samples were below WHO reference limits^33^.

**Table 2:** Distribution of sperm parameters (n = 500).

Semen parameters	Mean (SD)	Percentiles				
		5th	25th	50th	75th	95th
Routine parameters						
Sperm concentration (×10^6^/ml)	55.55 (48.4)	7.23	21.03	41.42	74.21	151.99
Total count (×10^6^)	111.79 (149.3)	7.01	24.86	60.94	129.66	406.15
Forward mobility (%)	41.96 (18.15)	10.05	29.67	43.37	54.19	70.28
Sperm velocities						
VCL (μm/s)	53.99 (11.76)	33.46	46.31	53.88	62.3	72.55
VAP (μm/s)	39.17 (9.25)	23.15	32.89	40.28	45.16	53.32
VSL (μm/s)	35.01 (9.03)	20.34	29.27	35.48	40.52	48.91
Velocity ratios						
LIN (%)	61.77 (8.83)	48.41	56.59	61.42	67.57	75.48
STR (%)	84.46 (6.17)	76.74	81.8	84.77	87.62	91.95
WOB (%)	70.09 (7.25)	59.71	66.76	71.02	75.95	82.9
Wobble characteristics						
ALH (μm)	3.33 (1.17)	1.37	2.58	3.34	4.09	5.17
MAD (degree)	56.55 (9.92)	39.8	49.94	56.35	63.85	71.87
BCF (Hz)	4.95 (1.13)	3.5	4.38	4.87	5.38	6.28

### Associations between urinary BPA and sperm parameters

**Figure 1:**
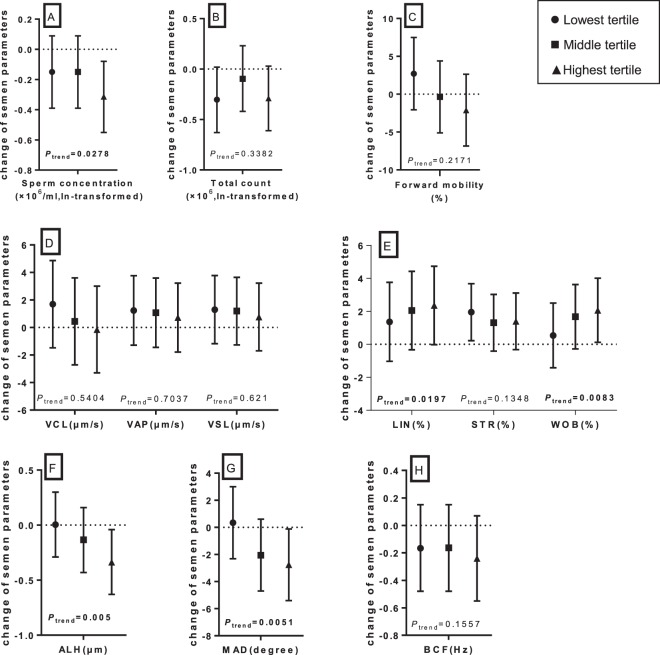
Adjusted dose-response relationship between urinary creatinine-adjusted BPA levels and semen parameters with a reference group of undetected BPA: sperm concentration (**A**), total count (**B**), forward mobility (**C**), sperm motion velocity (**D**), velocity ratio (**E**), Amplitude of lateral head displacement (**F**), Mean angular displacement (**G**), and Beat-cross frequency (**H**). With the reference of undetected BPA, the model included 132, 106, 109 and 110 men in undetected BPA group, lowest tertile, middle tertile and highest tertile of detected BPA groups, respectively; There are 43 missing values in creatinine measurements in BPA dectected group. Range of BPA tertiles (μg/gCr): lowest tertile, LOD-0.35; middle tertile, 0.36–1.29; highest tertile: >1.29; Adjusted for age, education, race, smoking, alcohol intake, BMI, abstinence period, history of pesticide usage and occupational exposure to high temperature.

Table 3 shows distributions of sperm parameters of subjects in undetected and detected BPA groups. A marginally statistically significant association was found of BPA with sperm concentration. Sperm count and forward mobility were also lower in BPA detected group, although the associations were not statistically significant. We observed statistically significant higher LIN, STR, and WOB and lower ALH and MAD in BPA detected group. Further analyses examining dose-responsive associations are illustrated by Fig. 1. Compared with subjects in undetected BPA group, subjects in group of the highest BPA tertile had significantly lower sperm concentration with a decreasing trend with BPA levels. LIN, STR, and WOB were higher in subgroups of detected BPA, and statistically significant increasing trends with BPA levels were presented in LIN and WOB, but not statistically significant in STR. In contrast, ALH, MAD, and BCF tended to decrease with increasing BPA levels, although the trend of BCF seemed not statistically significant.

**Table 3:** Associations between urinary BPA and sperm parameters (n = 500).

Sperm parameters	Mean in undetected BPA group	Mean in detected BPA group	Crude		Adjusted	
			β (95% CI)	*p*-value	β (95% CI)	*p*-value
Sperm concentration (×10^6^/ml, ln-transformed)	3.76	3.61	−0.14 (−0.33, 0.04)	0.1231	−0.15 (−0.34, 0.03)	0.1031
Total count (×10^6^, ln-transformed)	4.13	4.03	−0.11 (−0.35, 0.14)	0.3922	−0.14 (−0.39, 0.11)	0.2692
Forward mobility (%)	42.31	41.83	−0.48 (−4.1, 3.14)	0.7931	−0.78 (−4.48, 2.93)	0.6801
VCL (μm/s)	53.95	54	0.05 (−2.29, 2.4)	0.9647	−0.24 (−2.68, 2.19)	0.845
VAP (μm/s)	38.54	39.4	0.86 (−0.98, 2.71)	0.3591	0.55 (−1.36, 2.47)	0.57
VSL (μm/s)	34.29	35.27	0.98 (−0.82, 2.78)	0.2849	0.68 (−1.19, 2.55)	0.4779
LIN (%)	60.08	62.38	**2.29** (**0.54, 4.04)**	**0.0104**	**2.19** (**0.37, 4.0)**	**0.0184**
STR (%)	83.35	84.86	**1.51** (**0.29, 2.73)**	**0.0157**	**1.47** (**0.19, 2.75)**	**0.025**
WOB (%)	69.72	71.58	**1.86** (**0.43, 3.3)**	**0.0111**	**1.75** (**0.26, 3.25)**	**0.0217**
ALH (μm)	3.5	3.26	**−0.23** (**−0.47, −0.01)**	**0.0452**	**−0.26** (**−0.5, −0.02)**	**0.0334**
MAD (degree)	58.11	55.99	**−2.11** (**−4.09, −0.14)**	**0.0355**	**−2.17** (**−4.22, −0.11)**	**0.0391**
BCF (Hz)	5.13	4.89	**−0.25** (**−0.47, −0.02**)	**0.0314**	−0.21 (−0.45, 0.03)	0.0801

With the reference of undetected BPA, the model included 132 and 368 men in undetected and detected BPA groups respectively.

Adjusted for age, education, race, smoking, alcohol intake, BMI, abstinence period, history of pesticide usage and occupational exposure to high temperature.

### Sub-group analyses

When analyses were restricted to subjects whose urine samples were collected between 9 to 11 am or whose semen samples were obtained within an abstinence period of 2 to 7 days (Table 4), we observed larger estimates of associations between urinary BPA concentrations and sperm concentrations, sperm velocity ratios, and swing characteristics, although the sample sizes shrank remarkably. The enlargements of association estimates were more evident when time of urine collection was restricted.

**Table 4:** Associations between urinary BPA and semen parameters in subjects whose urine was collected during 9 to 10 am (model 1) and subjects whose semen samples were obtained within abstinence period of 2 to 7 days (model 2).

	Urine collected during 9 to 11 am (n = 135, model 1)		Abstinence period of 2 to 7 days (n = 282, model 2)	
	Adjusted β (95% CI)	*p*-value	Adjusted β (95% CI)	*p*-value
Sperm concentration (×10^6^/ml, ln-transformed)	**−0.36 (−0.71, −0.01)**	**0.0464**	−0.15 **(**−0.4, 0.09)	0.2186
Total count (×10^6^, ln-transformed)	−0.42 (−0.92, 0.07)	0.0914	−0.06 (−0.37, 0.26)	0.7194
Forward mobility (%)	1.12 (−7.1, 9.33)	0.7882	0.9 (−3.94, 5.74)	0.7148
VCL (μm/s)	2.35 (−2.77, 7.48)	0.3650	−0.12 (−3.28, 3.04)	0.9409
VAP (μm/s)	1.78 (−2.17, 5.73)	0.3730	0.78 (−1.7, 3.27)	0.5349
VSL (μm/s)	1.93 (−1.94, 5.8)	0.3244	1.04 (−1.39, 3.46)	0.4016
LIN (%)	3.74 (−0.39, 7.88)	0.0757	**2.92** (**0.61, 5.22)**	**0.0133**
STR (%)	**3.78** (**−0.02, 7.58)**	**0.0513**	**2.54** (**0.72, 4.35)**	**0.0064**
WOB (%)	2.53 (−0.9, 5.95)	0.1464	**2.04** (**0.17, 3.92)**	**0.0324**
ALH (μm)	0.18 (−0.36, 0.72)	0.5099	−0.09 (−0.38, 0.21)	0.5563
MAD (degree)	−3.61 (−8.18, 0.96)	0.1206	−2.0 (−4.62, 0.61)	0.1329
BCF (Hz)	**−0.61** (**−1.15, −0.06)**	**0.0311**	**−0.35** (**−0.63, −0.06)**	**0.0188**

With the reference of undetected BPA, model 1 included 35 and 100 men in undetected and detected BPA groups respectively and the corresponding numbers were 82 and 200 in model 2;

Adjusted for age, education, race, smoking, alcohol intake, BMI, abstinence period, history of pesticide usage and history of occupational exposure to high temperature 1;

Adjusted for age, education, race, smoking, alcohol intake, BMI, history of pesticide usage and occupational exposure to high temperature in model 2.

### Repeated analyses using unadjusted BPA concentrations

The associations between urinary BPA concentrations and sperm parameters and the corresponding dose-responsive relationships were confirmed when we repeated the analyses using unadjusted BPA concentrations of all subjects (Supplemental Material, Table S1).

## Discussion

The study was, to our knowledge, the first report to examine the relationships between exposure to environmental BPA and both routine sperm parameters and sperm movement characteristics in a relatively large sample of Chinese population. Environmental exposure to BPA was associated with lower sperm concentration, higher sperm velocity ratios (LIN, STR and WOB), and lower sperm swing characteristics (ALH and MAD). The associations were found to be dose-responsive except for STR.

Several epidemiological studies have examined the associations between BPA and routine sperm parameters. Lower sperm concentration, sperm count, and motility were reported to be associated with urinary BPA concentrations^16–18^, although the associations were not statistically significant in two studies^19,20^. The main findings of these studies accorded with our results to a large extent. In two studies^17,19^ measuring sperm movement characteristics, urinary BPA concentrations were reported to be associated with lower VCL in one study^17^, but this study recruited subjects from infertility clinics with relatively small sample size (190 men). The other study^19^ reported that BPA had no effects on sperm movement characteristics, but this study evaluated sperm parameters 24 hours later after ejaculation, which may have resulted in inaccurate assessment of time-sensitive parameters such as sperm movement characteristics and concealed the true associations.

It’s no doubt that semen quality is the dominant component determining male fertility, among which sperm concentration is closely linked to male fecundity and is a crucial component of semen analysis, the first step to identify male infertility^33,34^. CASA system is recommended by WHO because of its high precision and capacity to provide quantitative data on kinematic parameters of spermatozoa^33^. Current evidence of *in vitro* studies and human studies^21,22,24–26,35^ has shown that reduction of VCL and ALH, and an increase of LIN are related to poor male fertility. Defined by these specific sperm movement characteristics, hyperactivated mobility (HA), a vigorous pattern of sperm motility marked by wide-amplitude, high-velocity, whiplash movements of the flagellum, has been proved to be related to the functional competence of the spermatozoa to penetrate the zona pellucida^36^. Therefore, BPA-induced reductions of sperm concentration and ALH and increase of LIN observed in our study, might lead to impairment of male fertility. Although animal studies, in agreement with our findings, showed associations between BPA and male infertility^11,13,37^, two existing human studies^38,39^ disclosed that male BPA concentrations had no effects on pregnancy rates or time to pregnancy. Further research is needed to confirm the possible impairments of BPA on human fertility through changes of sperm movement patterns.

The mechanisms of the association between BPA and decreasing sperm concentration have been well documented. Studies have shown that BPA may target Sertoli cells and block the meiotic progression of germ cells, which in turn have a direct adverse impact on spermatogenesis^40^. In addition, BPA, acting as an androgen receptor (AR) antagonist, interrupts the normal AR binding activity and the interaction between AR and endogenous androgens^41^. Such an interruption by BPA on the function of endogenous androgens could conceivably interfere with normal spermatogenesis. However, studies revealing mechanisms of BPA changing sperm movement patterns are sparse. Experimental studies have shown that BPA inhibits the sperm mobility through compromising mitochondrial functions and decreasing ATP levels in spermatozoa^13,37,42^, and in turn decreases male fertility. BPA-induced decreases in sperm motility could also be explained by the increase of mitochondrial ROS and the reduction of high mitochondrial inner trans-membrane potential^10^. However, mechanisms in more specific pathways of interest are needed to be investigated and disclosed.

Our findings offer new insights with regard to reduction of sperm concentration and change of sperm movement characteristics in relation to BPA exposure. Especially, our study recruited subjects from a general and fertile population, rather than from clinical or occupational settings, a community-based nature of study design. The study site of our study, Sandu county of Guizhou Province, is a remote and less industrialized area of China. The GM of urinary BPA concentration of our study population (0.38 μg/L) was lower than three previous reports in general Chinese populations (0.87 μg/L^3^, 1.01 μg/L^29^ and 2.23 μg/L^5^). Our findings are informative about environmental BPA exposure at a lower level and should be brought to the forefront since obvious inverse associations were found. Assessments of sperm parameters performed by the same professional technician using CASA system within 1 hour following ejaculation ensured the accuracy of the measurement of these time-sensitive sperm movement characteristics.

Our study should also be interpreted in light of limitations. Firstly, we selected only fertile men and caution should be taken when generalizing the results to other population. Secondly, a single urine sample but not the first morning voids was collected to assess BPA exposure. BPA is rapidly metabolized within several hours in humans. Therefore, the single urine sample may only reflect the most recent BPA exposure. However, a study^43^ found that when population investigated is sufficiently large and samples are randomly collected relative to meal ingestion times and bladder emptying times, the single spot-sampling approach may adequately reflect the average exposure of the population to BPA. Moreover, another study^44^ showed that a single-spot urine sample could predict a subject’s tertile categorization with moderate sensitivity. The third limitation is the large discrepancy in urine collection time which might lead to misclassifications of exposure measures. However, any variation in the measurement of BPA exposure would have resulted in non-differential misclassification of BPA exposure levels that is independent of outcomes. Existence of such misclassification would have resulted in under-estimation of the strength of the observed associations^45^. Besides, two sensitivity analyses were performed to corroborate our findings. When analyses were limited, respectively, to subjects whose urine samples were collected during 9 to 11 am and whose semen samples were obtained within an abstinence period of 2 to 7 days, stronger associations were observed, as anticipated, compared with the overall analyses. These results further strengthened our findings. Fourthly, BPA was measured in urine but not seminal fluid. Because BPA is relatively non-persistent (biological half-life < 6 hours) and a sufficient amount of it undergoes rapid excretion in urine, urinary measurements of BPA are most preferred in estimating human uptake or exposure^46^. Moreover, seminal BPA only works in the late stage of spermatogenesis when spermatozoa enters epididymis. However, investigation on toxic effects of seminal BPA is interesting and valuable in further studies to reveal mechanisms. Finally, BPA was measured by HPLC rather than lipid chromatography/mass spectrometry (LC-MS/MS), and BPA levels in the HPLC analysis were generally lower than those in LC-MS/MS methods^47^. However, a study conducted by Bitna Yi^47^, which measured BPA concentrations in human breast milk using both LC-MS/MS and HPLC, found that there were moderate associations between total BPA levels with the two methods (R^2^ = 0.40, p < 0.01). Although possible over-estimation with the LC-MS/MS method in low BPA samples could not be ruled out^47^, a segment of undetectable samples by HPLC could be detected by LC-MS/MS^48^. However, the misclassification of BPA exposure may bias the results toward null but not overturn the statistically significant associations.

## Conclusion

In conclusion, environmental exposure to BPA in a less industrialized area of China, where human urine BPA level is relatively lower, is associated not only with decreased sperm concentration, but also with increased sperm velocity ratios (LIN, STR and WOB), and reduced sperm swing characteristics (ALH and MAD). Impaired spermatogenesis and sperm movement may explain male subfertility resulting from exposure to BPA, although the biological mechanism is still uncertain and, therefore, needs to be disclosed by future studies.

## Electronic supplementary material

Supplemental Materials

## Data Availability

The datasets generated and/or analysed during the current study are not publicly available due to subject confidentiality but are available from the corresponding author on reasonable request.
