# Supplementary material for: Exposure of environmental Bisphenol A in relation to routine sperm parameters and sperm movement characteristics among fertile men
Source: Sci Rep. 2018 Dec 3;8:17548. doi: 10.1038/s41598-018-35787-5 (PMC6277384; doi:10.1038/s41598-018-35787-5)
Supplement: Supplementary file 1 — Supplemental Materials [file 41598_2018_35787_MOESM1_ESM.docx]

**Supplemental Materials**

**Exposure of environmental Bisphenol A in relation to routine sperm parameters and sperm movement characteristics among fertile men**

Honglei Ji, Maohua Miao, Hong Liang, Huijuan Shi, Dasheng Ruan, Yongbo Li, Jian Wang, Wei Yuan

Supplemental Table S1. Associations between unadjusted urinary bisphenol A (BPA) and semen parameters (n=500)

Supplemental Table S2. Distribution of urinary BPA concentration according to urine collection time in a day

**Supplemental Table S1. Associations between unadjusted urinary bisphenol A (BPA) and semen parameters (n=500)**

|  | **BPA detection** | **Mean** | **Adjusted β (95%CI)** | **P-trend** |
| --- | --- | --- | --- | --- |
| Sperm concentration  (×10^6^/ml, ln-transformed) | No | 3.76 | Ref |  |
|  | Lowest tertile | 3.74 | -0.05 (-0.29, 0.19) | **0.0242** |
|  | Middle tertile | 3.59 | -0.14 (-0.37, 0.08) |  |
|  | Highest tertile | 3.52 | **-0.25 (-0.48, -0.02)** |  |
| Total count (×10^6^, ln-transformed ) | No | 4.13 | Ref |  |
|  | Lowest tertile | 4.05 | -0.16 (-0.48, 0.16) | 0.338 |
|  | Middle tertile | 4.05 | -0.09 (-0.39, 0.21) |  |
|  | Highest tertile | 3.97 | -0.17 (-0.48, 0.13) |  |
| Forward mobility (%) | No | 42.31 | Ref |  |
|  | Lowest tertile | 46.05 | 2.18 (-2.59, 6.95) | 0.1621 |
|  | Middle tertile | 40.14 | -1.58 (-6.06, 2.89) |  |
|  | Highest tertile | 39.91 | -2.41 (-6.98, 2.16) |  |
| VCL (μm/s) | No | 53.95 | Ref |  |
|  | Lowest tertile | 55.0 | 0.03 (-3.12, 3.18) | 0.755 |
|  | Middle tertile | 53.75 | -0.3 (-3.25, 2.66) |  |
|  | Highest tertile | 53.38 | -0.41 (-3.43, 2.61) |  |
| VAP (μm/s) | No | 38.54 | Ref |  |
|  | Lowest tertile | 39.74 | 0.38 (-2.09, 2.86) | 0.5939 |
|  | Middle tertile | 39.44 | 0.69 (-1.63, 3.01) |  |
|  | Highest tertile | 39.05 | 0.55 (-1.82, 2.93) |  |
| VSL (μm/s) | No | 34.29 | Ref |  |
|  | Lowest tertile | 35.59 | 0.54 (-1.88, 2.95) | 0.5073 |
|  | Middle tertile | 35.27 | 0.73 (-1.54, 3.0) |  |
|  | Highest tertile | 34.98 | 0.73 (-1.58, 3.05) |  |
| LIN (%) | No | 60.08 | Ref |  |
|  | Lowest tertile | 61.72 | 1.65 (-0.69, 4.0) | **0.0243** |
|  | Middle tertile | 62.75 | **2.41 (0.2, 4.61)** |  |
|  | Highest tertile | 62.56 | **2.4 (0.15, 4.65)** |  |
| STR (%) | No | 83.35 | Ref |  |
|  | Lowest tertile | 85.05 | **1.67 (0.01, 3.33)** | 0.0751 |
|  | Middle tertile | 84.64 | 1.17 (-0.38, 2.73) |  |
|  | Highest tertile | 84.92 | **1.61 (0.02, 3.2)** |  |
| WOB (%) | No | 69.72 | Ref |  |
|  | Lowest tertile | 70.69 | 1.01 (-0.92, 2.94) | **0.016** |
|  | Middle tertile | 72.1 | **2.13 (0.32, 3.94)** |  |
|  | Highest tertile | 71.82 | **1.99 (0.13, 3.84)** |  |
| ALH (μm) | No | 3.5 | Ref |  |
|  | Lowest tertile | 3.39 | -0.16 (-0.47, 0.14) | **0.0299** |
|  | Middle tertile | 3.18 | **-0.3 (-0.59, -0.01)** |  |
|  | Highest tertile | 3.24 | -0.29 (-0.59, 0.003) |  |
| MAD (degree) | No | 58.11 | Ref |  |
|  | Lowest tertile | 57.92 | -0.63 (-3.28, 2.02) | **0.0135** |
|  | Middle tertile | 54.84 | **-3.0 (-5.48, -0.51)** |  |
|  | Highest tertile | 55.53 | **-2.58 (-5.12, -0.04)** |  |
| BCF (Hz) | No | 5.13 | Ref |  |
|  | Lowest tertile | 4.98 | -0.13 (-0.43, 0.18) | 0.103 |
|  | Middle tertile | 4.81 | **-0.30 (-0.58, -0.01)** |  |
|  | Highest tertile | 4.88 | -0.19 (-0.49, 0.1) |  |

The model included 132, 110, 135 and 123 men in undetected BPA group, lowest tertile, middle tertile and highest tertile of detected BPA groups, respectively;

Range of BPA tertiles (μg/L): lowest tertile, LOD-0.31; middle tertile, 0.32-1.12; highest tertile, >1.12;

Adjusted for age, education, race, smoking, alcohol intake, BMI, abstinence period, history of pesticide usage and history of occupational exposure to high temperature.

**Supplemental Table S2. Distribution of urinary BPA concentration according to urine collection time in a day**

|  | **unadjusted (μg/L)**  **n=500** | | **creatinine-adjusted (****μg/gCr)**  **n=448** | |
| --- | --- | --- | --- | --- |
|  | **N** | **Mean** | **N** | **Mean** |
| 7 am | 12 | 0.381 | 12 | 0.342 |
| 8 am | 64 | 0.895 | 59 | 1.578 |
| 9 am | 73 | **4.913** | 73 | **6.711** |
| 10 am | 62 | **2.357** | 59 | **2.957** |
| 11 am | 50 | 1.837 | 47 | 2.111 |
| 12 am | 18 | 1.162 | 17 | 1.628 |
| 1 pm | 52 | 1.617 | 52 | 1.788 |
| 2 pm | 24 | 0.591 | 22 | 0.65 |
| 3 pm | 36 | 1.804 | 35 | 1.702 |
| 4 pm | 18 | 1.202 | 17 | 1.325 |
| 5 pm | 24 | 0.959 | 24 | 1.739 |
| 6 pm | 6 | 0.369 | 6 | 0.736 |
| missing | 61 | 1.129 | 25 | 2.297 |
